# The beneficial cardiovascular effects of bariatric surgery are similar to dietary weight loss in obesity

**DOI:** 10.1186/1532-429X-11-S1-P139

**Published:** 2009-01-28

**Authors:** Oliver J Rider, Jane M Francis, Matthew D Robson, Monique Robinson, Steffen E Petersen, James P Byrne, Stefan Neubauer

**Affiliations:** University of Oxford, Oxford, UK

**Keywords:** Bariatric Surgery, Right Ventricular, Cardiovascular Magnetic Resonance, Aortic Distensibility, Percentage Excess Weight Loss

## Objective

Our aim was to investigate the beneficial cardiovascular effects of bariatric surgery versus dietary weight loss in obesity.

## Background

Bariatric surgery confers better long term weight management than dietary intervention, is an increasingly utilized method of weight management, and has been shown to reduce mortality. Despite this, no study to date has addressed the relative beneficial effects of these different weight loss approaches on cardiac and aortic structure and function. In view of this, we used cardiovascular magnetic resonance imaging (CMR) to compare the effect of either bariatric surgery or dietary weight loss on left (LV) and right ventricular (RV) structure, LV diastolic function and regional aortic elastic function in a group of healthy obese subjects.

## Methods

30 obese subjects, with no identifiable cardiovascular risk factors were recruited to the study. All subjects underwent cardiac MR imaging at 1.5 T for the assessment of left ventricular mass (g), left ventricular end-diastolic volume (EDV; ml), stroke volume (SV; ml) and LV EF (%). Aortic distensibility (AD) was assessed at three levels; the ascending (Ao) and proximal descending aorta (PDA) at the level of the pulmonary artery and the abdominal aorta (AA) The abdominal cine images were piloted perpendicular to the orientation of the abdominal aorta. In addition to this left ventricular diastolic function was assessed using volume time curve analysis.

CMR was performed at pre and post weight loss intervention, in 17 subjects on GI index diet (BMI 35.2 ± 5.5) and 13 subjects undergoing Bariatric surgery (BMI 45.6 ± 6.0 kg/m^2^).

## Results

Both groups achieved significant weight loss, which tended to be greater after Bariatric surgery (p = 0.32). Fasting serum glucose, cholesterol, systolic & diastolic blood remained within the normal range for both groups before and after intervention. Both groups had significant improvements in LV & RV mass, LV & RV EDV, diastolic filling rate and abdominal AD. When the improvements were normalized to percentage excess weight loss, there was no significant difference in the beneficial effects achieved in the surgical group to those seen in the dietary group for any of the cardiovascular parameters (Table 1).

**Table 1:** Table 1

Absolute Values		Normalized to % Excess Weight Loss (× 10^-2^)	
Bariatric Cohort (N = 13)	Diet Cohort (N = 17)	Bariatric Cohort (N = 13)	Diet Cohort (N = 17)
61 ± 22	48 ± 42	_____	_____
19 ± 12*	10 ± 11*	2.7 ± 3.7	2.9 ± 2.5
13 ± 14*	9 ± 9*	1.7 ± 3.2	3.4 ± 4.2
25 ± 12*	19 ± 8*	6.9 ± 5.1	4.8 ± 3.4
4 ± 17	10 ± 18	0.3 ± 3.2	4.8 ± 10
1.2 ± 2.7	0.7 ± 2.3	2.0 ± 4.0	3.0 ± 11
1.9 ± 1.9*	0.1 ± 1.5*^#^	3.0 ± 3.0	1.0 ± 6.0
1.5 ± 2.0*	1.8 ± 1.8*	3.0 ± 3.0	7 ± 10
0.78 ± 0.77*	0.77 ± 0.84*	0.14 ± 1.0	2.0 ± 3.0

* = p < 0.05 Pre vs Post Weight Loss # = p > 0.05 Surgical Vs Dietary Weight Loss

## Conclusion

Irrespective of method, weight loss results in beneficial cardiovascular effects. Bariatric surgery and dietary weight loss provide similar levels of improvement in cardiovascular structure and function.

